# A Rare Case of Orbitocranial Penetrating Injury with Intracranial Wooden Foreign Body Residue

**DOI:** 10.3390/medicina58121832

**Published:** 2022-12-12

**Authors:** Yi Wu, Weimin He, Yiliu Yang, Jun Chen

**Affiliations:** Department of Ophthalmology, West China Hospital, Sichuan University, No 37 Guoxue Street, Chengdu 610041, China

**Keywords:** wooden foreign body, orbitocranial penetrating injury, intracranial injury, unilateral subfrontal approach

## Abstract

Orbitocranial penetrating injuries (OPIs) caused by wooden foreign bodies (WFBs) are very rare and life threatening. Their diagnosis and treatment could be challenging for an ophthalmologist, requiring us to remain alert for possible intracranial extension. We present a case of a 52-year-old man with a residual wooden foreign body in the left frontal lobe. He had a notable history of trauma from a fall on a tree stump and surgical removal of a wooden foreign body from his left orbit 6 years ago. He was referred to us due to recurrent discharge from the eyelid wound. Wooden foreign body residue was successfully removed with a carefully planned craniotomy without complications. This case describes the clinical manifestation, radiographic features, and treatment of this rare trauma, with an emphasis on imaging diagnosis and multi-disciplinary management.

## 1. Introduction

Orbitocranial penetrating injuries (OPIs) are relatively uncommon in clinical practice, accounting for 24% of penetrating head traumas in adults and 45% in children [1]. The cause of injury could be metal, glass, wood, or plastic objects. Nevertheless, transorbital penetrating intracranial injuries by wooden foreign bodies (WFBs) are exceedingly rare. Only a handful of cases have been reported worldwide [2,3,4,5]. The penetration is typically through the superior orbital fissure into the cavity because of the conical shape of the orbit [6]. Neurological symptoms may not manifest immediately at the time of injury, which could result in a delay in diagnosis and severe complications. If not completely removed, the WFB residue in the orbit and/or cranial cavity could cause a serious infection. In this case report, we present the first case of an OPI with a residual intracranial WFB in 6 years. We describe the clinical course, imaging features, and treatment before briefly reviewing the relevant literature.

## 2. Case Report

A 52-year-old man fell and hit his left eye on a tree stump 6 years ago. A WFB penetrated the left upper eyelid and extended into the left orbit. The patient remained conscious after the incident and was taken to the local hospital; then, an emergency computed tomography (CT) scan of the orbit revealed a foreign body in the orbit without any involvement of the eyeball. Subsequently, the WFB was removed from the left orbit and the skin was sutured in emergency surgery before antibiotics were given. Unfortunately, the physician was unaware of an intracranial foreign body. The patient began to have recurrent pus discharged from the wound 4 months after injury, without visual impairment, ocular motility limitation, neurological abnormalities, or a fever. Subsequent CT scanning failed to reveal any residual foreign bodies. The patient was given a one-time pus drainage without further treatment.

**Figure 1:**
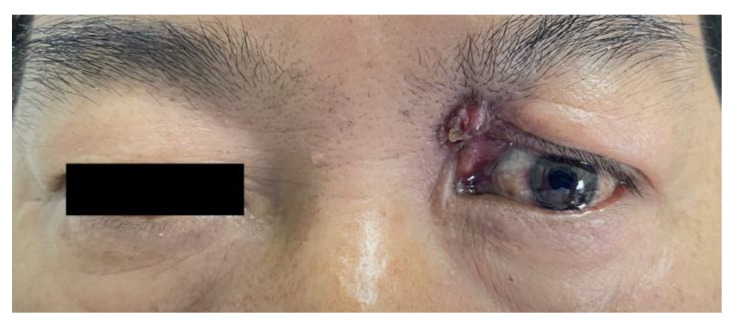
A scar contracture was seen on the medial side of the left upper eyelid.

**Figure 2:**
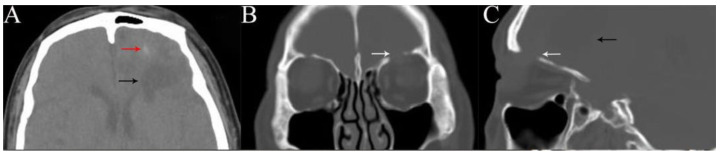
Head computed tomography (CT) scan showed a short, strip-like, slightly high-density shadow (red arrow) and a cystic mass (black arrow) in the left frontal lobe, and a fracture of the left superior orbital wall (white arrow). Axial (**A**), coronal (**B**), and sagittal views (**C**).

**Figure 3:**
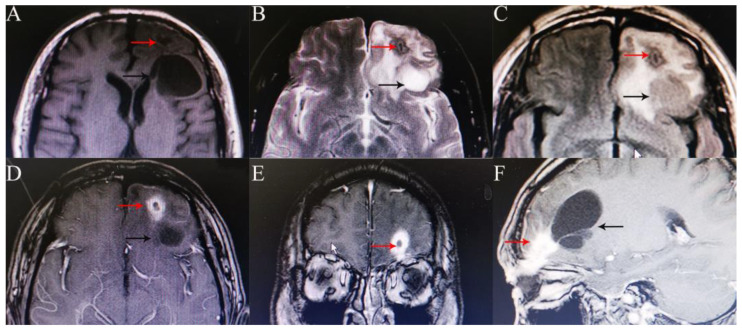
Head magnetic resonance imaging (MRI) showed a strip-shaped lesion that stretched from the left orbital roof to the left frontal lobe, was isointense on the T1-weighted image, slightly hyperintense on the T2-weighted image, and isointense/hypointense on FLARE (**A**–**C** red arrow), with obvious irregular enhancement surrounding it (**D**–**F** red arrow), and a 3.9 × 3.4 × 4.9 cm cystic shadow behind the lesion (black arrow).

He was referred to the Department of Ophthalmology, West China Hospital, Sichuan University, for recurrent purulence of his left eyelid for 6 years. His best corrected visual acuity was 20/20 in both eyes. Cicatricial contracture of medial skin was seen in the left upper eyelid with lagophthalmos and pus flowing from the wound (Figure 1). The left eye globe was normal. His body temperature was normal. He was neurologically intact with normal muscle strength and tension. Routine blood tests showed no significant abnormalities. A head CT scan revealed a short, strip-like, slightly high-density shadow and a cystic mass (about 3.6 cm in length) in the left frontal lobe and an old fracture of the left superior orbital wall (Figure 2). A magnetic resonance imaging (MRI) of the head visualized a strip-shaped lesion that stretched from the left orbital roof to the left frontal lobe, was isointense on the T1-weighted image and slightly hyperintense on the T2-weighted image, with obvious irregular enhancement surrounding it.. A 3.9 × 3.4 × 4.9 cm cystic shadow was seen behind the lesion. The above imaging findings indicated a residual intracranial foreign body and a chronic infection (Figure 3).

An urgent neurosurgical intervention was sought and he was transferred to the Neurosurgery Department. The patient underwent a craniotomy with the subfrontal approach, where the WFB residue was completely removed with the surrounding mechanized brain tissue excised. Anti-infection therapy with broad-spectrum antibiotics (ceftriaxone sodium, 2 g × 7 days), anti-epilepsy therapy with sodium valproate (800 mg × 7 days), and brain nutritional drugs were administered after surgery. A head CT scan was obtained on the first postoperative day, which showed postoperative changes but no evidence of a residual foreign body or intracranial hematoma. The patient was discharged 7 days later without neurological deficits or cerebrospinal fluid leakage.

## 3. Discussion

An OPI caused by a WFB may invade the cranial space and result in fatal complications, such as brain damage, hemorrhage, and infection. Most OPI patients are adult males injured in a fall [7]. Other less frequently reported mechanisms include traffic accidents [2], animal kicks [3], fights [4], and physical assaults [5]. Children are typically injured while playing in school or at home. The penetrating WFB is not as hard as a metal or glass foreign body and the apex of the orbit is a pyramid-shaped structure; therefore, it is typically directed intracranially through the superior orbital fissure, the optic canal, or the thin orbital roof [6,8]. The superior orbital fissure is relatively common in all reported cases, which may affect the optic nerve, cavernous sinus, carotid artery, and even the brain stem. Other reported entry sites include the squamous portion of the temporal bone [9]. In our case, the patient had a WFB that penetrated through the orbital roof when he hit his head on a tree stump in a fall.

OPI patients may be referred to the ophthalmology department or emergency department when injured, especially when they have no neurological symptoms; therefore, thorough ophthalmologic, neurologic, and imaging examinations are required. The possibility of foreign body presence and intracranial injury, even in the absence of a visible skin wound, global injury, or neurological symptoms at the time of injury, should be carefully investigated. If a patient has persistent infections after trauma, a deep retained foreign body could be considered. As is alarming in our case, the patient’s intracranial foreign body residue remained undetected for up to 6 years after his initial injury and surgical removal. An orbitocranial sinus tract with recurrent purulency was formed, though without fatal complications.

Radiographic studies are essential for determining the shape, size, and trajectory of the foreign body penetration, supporting correct diagnosis, and choosing a proper surgical protocol. However, the organic components of a WFB might hinder the display of the fragment morphology and lead to misdiagnosis [10,11]. Dry wood often mimics pneumocephalus on a CT scan, which could be differentiated by its linear appearance and different attenuation. It may start to appear like soft tissue as the wood absorbs water over time [12]. As a result, CT has limitations in detecting WFBs. It is suggested that CT has a 42% missing rate for non-metallic foreign bodies. Multiple cases have been reported in which CT failed to identify an orbitocranial WFB [13,14]. Similarly, it is not easy for CT to identify a retained wooden foreign body. As time goes on, the increase in the density of wooden objects may be due to granulomas around foreign bodies or calcium deposits within the wood [15,16]. In contrast, MRI is a more sensitive diagnostic technology because dry wood is hypointense on the T1- and T2-weighted sequences, wet wood is hypointense on the T1-weighted and hyperintense on the T2-weighted scans, and gadolinium enhancement may reveal any inflammation of the surrounding soft tissues [12], as was the case in our case. Angiography is always necessary for any penetrating brain injury to exclude cerebrovascular injury and investigate the relationship between the foreign body and intracranial vascular structures [17,18]. This was conducted in most cases described in the literature. Meanwhile, follow-up CT angiography, MR angiography, or digital subtraction angiography are also recommended to rule out the possibility of a delayed cavernous carotid fistula and post-traumatic intracranial aneurysm formation [18].

There is no standard treatment for an OPI caused by a WFB. Nonetheless, wood can be a welcoming medium for bacteria because of its porosity and immediate contact with soil. This increases the risk of infection as well as panophthalmitis, and orbital and intracranial abscesses. Furthermore, wood can be fragile and easily broken when it penetrates or is being removed [19]. So, timely and complete removal of the WFB is the priority. Under the premise of angiography, if penetration of a hardened/processed WFB involves the cavernous sinus without damaging the internal carotid artery (ICA) or involves the intracranial space without damaging the eloquent areas and the distal end of the WFB protrudes from the skin, it can be pulled out with ICA proximal control in the operating theatre by neurosurgeons. However, the position or angle of the WFB may change when being extracted and damage the surrounding structures [4,20,21], although a few authors believe that direct removal of foreign bodies using broad-spectrum anticonvulsants is less dangerous and destructive than a craniotomy approach [22]. In contrast, if the WFB is soft and fragile, there is the involvement of the eloquent areas, ICA or other major vessels, or an intracranial abscess or hematoma is suspected, a craniotomy is indicated. We prefer the latter because it allows direct vision, resulting in the complete exposure of the foreign body and its surrounding neurovascular structures, and it also could control intraoperative accidents in time [23]. After the WFB is extracted, postoperative imaging is necessary to confirm the absence of the residual foreign body, abscess, hematoma, and edema. Broad-spectrum antibiotic therapy should be administered after surgery, often using a third-generation cephalosporin and vancomycin. If culture results are available, antibiotics can be adjusted. Anticonvulsant therapy for up to 7 days is recommended for preventing early seizures [24]. In our case, the patient was urgently transferred to the Neurosurgery Department because of his chronic infection due to extended retention of the WFB in the frontal lobe. He underwent a craniotomy where the WFB was removed under direct vision.

## 4. Conclusions

In cases of OPI, physicians must be alert to possible intracranial injuries and order thorough examinations. MRI is a superior diagnostic technique compared with CT in detecting a WFB and determining its relation with the surrounding tissues. The WFB must be completely removed as soon as possible. A multidisciplinary surgical team is recommendable, which should consist of an ophthalmologist, a neurosurgeon, and other specialists if necessary.

## Data Availability

The datasets used and/or analyzed in the current study are available from the corresponding author upon reasonable request.

## References

[1] Kasamo S., Asakura T., Kusumoto K., Nakayama M., Kadota K., Atsuchi M., Yamamoto Y. (1992). Transorbital penetrating brain injury. No Shinkei Geka.

[2] Dudani A., Pawar H., Dudani A.A., Dudani K., Dudani A. (2019). A challenging case of a large orbitocranial wooden foreign body in a child. Indian J. Ophthalmol..

[3] Dunn I.F., Kim D.H., Rubin P.A., Blinder R., Gates J., Golby A.J. (2009). Orbitocranial wooden foreign body: A pre-, intra-, and postoperative chronicle: Case report. Neurosurgery.

[4] Sun G., Yagmurlu K., Belykh E., Lei T., Preul M.C. (2016). Management Strategy of a Transorbital Penetrating Pontine Injury by a Wooden Chopstick. World Neurosurg..

[5] Chehade L.K., Curragh D., Selva D. (2019). Traumatic intraorbital wooden foreign body: Lessons learnt. Clin. Exp. Ophthalmol..

[6] Balasubramanian C., Kaliaperumal C., Jadun C.K., Dias P.S. (2009). Transorbital intracranial penetrating injury-an anatomical classification. Surg. Neurol..

[7] Arslan M., Eseoğlu M., Güdü B.O., Demir (2012). Transorbital orbitocranial penetrating injury caused by a metal bar. J. Neurosci. Rural Pract..

[8] Harsh G.R., Wilson C.B. (1984). Central nervous system mesenchymal chondrosarcoma. Case report. J. Neurosurg..

[9] Tabibkhooei A., Aslaninia A., Anousha K. (2019). Childhood Transorbital Skull Base Penetrating Injury: Report of 2 Cases and Review of Literature. World Neurosurg..

[10] Cho W.K., Ko A.C., Eatamadi H., Al-Ali A., Abboud J.P., Kikkawa D.O., Korn B.S. (2017). Orbital and Orbitocranial Trauma From Pencil Fragments: Role of Timely Diagnosis and Management. Am. J. Ophthalmol..

[11] Kudo S., Takei T. (2016). Computed tomography settings for optimal detection of wooden foreign bodies. Am. J. Emerg. Med..

[12] Dalley R.W. (1995). Intraorbital wood foreign bodies on CT: Use of wide bone window settings to distinguish wood from air. Am. J. Roentgenol..

[13] Wilson M.H., Collins T.R., Revington P.J. (2016). Orbitocranial wooden foreign body retrieved by transcranial and superior orbitotomy. Br. J. Oral Maxillofac. Surg..

[14] Maruya J., Yamamoto K., Wakai M., Kaneko U. (2002). Brain abscess following transorbital penetrating injury due to bamboo fragments--case report. Neurol. Med. Chir..

[15] Amano K., Kamano S. (1982). Cerebellar abscess due to penetrating orbital wound. J. Comput. Assist. Tomo..

[16] Braun J., Gdal-On M., Goldsher D., Borovich B., Guilburd J.N. (1987). Traumatic carotid aneurysm secondary to cavernous sinus penetration by wood: CT features. J. Comput. Assist. Tomo..

[17] Liu H.C., Qiu E., Zhang T.M., Zhao J.W., Song W.X., Fu J.D. (2008). Neurosurgical therapy of transorbital intracranial foreign bodies: Review of 28 cases. Zhonghua Yi Xue Za Zhi.

[18] du Trevou M.D., van Dellen J.R. (1992). Penetrating stab wounds to the brain: The timing of angiography in patients presenting with the weapon already removed. J. Neurosurg..

[19] Hamilton A., Meena M., Lawlor M., Kourt G. (2014). An unusual case of intraorbital foreign body and its management. Int. Ophthalmol..

[20] Xu F., Li J., Sun S., Guo E., Hao S., Hou Z., Leung G.K., Liu B. (2013). The surgical management of a penetrating orbitocranial injury with a bakelite foreign body reaching the brain stem. Brain Inj..

[21] Gupta S.K., Umredkar A.A. (2012). Juxtapontine abscess around a retained wooden fragment following a penetrating eye injury: Surgical management via a transtentorial approach. J. Neurosurg-Pediatr..

[22] Miller C.F., Brodkey J.S., Colombi B.J. (1977). The danger of intracranial wood. Surg. Neurol..

[23] Del Verme J., Giordan E., Marton E., Zanata R., Di Paola F., Canova G., Longatti P. (2020). Classification of orbitocranial wooden foreign body penetration injuries: What to do when they violate the intracranial space? A systematic review. J. Neurosurg. Sci..

[24] Zyck S., Toshkezi G., Krishnamurthy S., Carter D.A., Siddiqui A., Hazama A., Jayarao M., Chin L. (2016). Treatment of Penetrating Nonmissile Traumatic Brain Injury. Case Series and Review of the Literature. World Neurosurg..

